# Conflict or Coordination? Spatiotemporal Coupling of Urban Population–Land Spatial Patterns and Ecological Efficiency

**DOI:** 10.3389/fpubh.2022.890175

**Published:** 2022-05-17

**Authors:** Ling Shan, Yuehua Jiang, Cuicui Liu, Jing Zhang, Guanghong Zhang, Xufeng Cui

**Affiliations:** School of Business Administration, Zhongnan University of Economics and Law, Wuhan, China

**Keywords:** ecological efficiency, coupling, Hubei Province, China, urbanization

## Abstract

The coordinated relationship between urban population–land spatial patterns (UPLSPs) and ecological efficiency (EE) is conducive not only to the rational utilization of resources and environment and the sustainable development of society, but also to the provision of a living environment that benefits public health. Identifying the coupling relationship of urban development and EE can provide critical information for urban planning. Previous studies have mainly focused on the coupling relationship between urban population and land, urbanization, and ecological development, while ignoring that between UPLSPs and EE. This study integrates several models to construct a novel framework for coupling UPLSPs and EE. Taking Hubei Province as the research area, we calculate the UPLSPs, EE, and their coupling coordination degree for 12 cities from 2000 to 2019. The paper offers several conclusions. (1) the urban population–land spatial matching degree increased, but the overall matching level was not high; the average value of EE showed an “N”-shaped change trajectory, and its overall level was low, with small changes and obvious regional differences. (2) The average value of the coupling coordination degree between UPLSPs and EE was a slow upward trend, with a radial distribution high in the middle and low in the periphery. There was conflict between the spatial patterns and EE, and the former restricted the development of the latter. (3) There were strong correlations between coordination degree and various indicators of UPLSPs and EE. While we should revitalize the stock of construction land and optimize the upgrading of the industrial structure, we also must coordinate human and land resources and the ecological environment, and narrow regional development differences. This study provides a new framework for urban environmental assessment and urban planning decision-making.

## Introduction

Urbanization has become an important trend in global development (1). Currently, 55% of the world's population lives in urban areas, a proportion that is expected to increase to 68% by 2050. Overall growth could add another 2.5 billion people to urban areas by 2050, with close to 90% of this increase taking place in Asia and Africa (2). China has experienced the largest and fastest urbanization process in the history of the world. At the end of 2019, China's urbanization rate reached 60.60%, an increase of 49.96 percentage points since 1949, and an average annual increase of 0.71 percentage points (3). Urbanization is undoubtedly an important driving force for China's economic development (4). However, the rapid development of cities has also spawned some inevitable problems, such as the reduction of biodiversity, the unreasonable allocation of urban resources, deterioration of the ecological environment, and urban emergencies (5–10), and so on, which have placed enormous pressure on China's public health, social economy, resources, and environment. To solve these problems, it is necessary to guide urban construction with the concept of ecological civilization, and to coordinate the relationship between urban development and ecological efficiency (EE).

The most important aspects of urbanization are population and land (11, 12). The urban population–land spatial patterns (UPLSPs) is the form of spatial agglomeration of urbanization elements. EE is the efficient use of ecological resources to meet human needs. It involves not only the unity of economic benefits and resources with environmental benefits, but also the essential embodiment of green and sustainable development (13). As an important grain production base and ecological barrier in the central region, Hubei Province has a superior location and a strong economy. It is a strategic support in the rise of central China. However, the contradictions between population agglomeration, urban land expansion, and ecological environment and economic development in this region have become increasingly prominent, threatening public health and the sustainable development of the city. How to deal with the relationship between the UPLSPs and EE and realize the rational utilization of resources and the sustainable development of society is an important task. What is the relationship between the UPLSPs and EE in Hubei Province? Is it one of conflict or coordination? These issues deserve in-depth study.

The concept of EE was first put forward by Schaltegger and Sturm in (14), and its original meaning was the ratio of economic value added to environmental impact. Subsequently, the World Business Council for Sustainable Development expanded the meaning to include obtaining products and services that could meet the needs of human life at the cost of minimizing resource consumption and environmental impact. At present, much research on urbanization and EE has been carried out, but these studies mainly focus on a single dimension of the topic. Most scholars believe that current urbanization features uncoordinated growth and is excessive (15). Relevant research mainly focuses on three aspects: (1) the spatial distribution differences of urban populations and land (16); (2) the calculation of coupling coordination degree between urban populations and land; and (3) uncoordinated factors of urban populations and land. Moreover, research on the coordination between population and land urbanization uses the coupling coordination model (17–20), the elastic coefficient model (21), and the Tapio decoupling model (22), among others. Scholars have proposed that location factors, economic development level, household registration systems, and land financialization (23, 24) all have important impacts on urban populations and land.

Moreover, academic research on EE has been growing and mainly focuses on three aspects. First, some studies explore the measurement index system and method of EE, mainly by using measurement index systems, such as “input + desirable output + undesirable output” (25, 26) and “resource consumption input + environmental impact input + output” (27, 28). Scholars also use the data envelopment analysis (DEA) method (29–31), slacks-based measure (SBM) model (32, 33), stochastic frontier approach (34, 35), Malmquist productivity index approach (36, 37), and ecological footprint model (38, 39) to calculate EE; among these, the DEA method and the SBM model are the most widely used. Second, scholars have analyzed evolution in the spatiotemporal patterns of EE across regions using methods such as spatial autocorrelation, Markov chain models, and the Theil index (40, 41). Third, research has applied Tobit models, spatial econometric models, system GMM methods, and other approaches to study the effects of urbanization, technological innovation, industrial structure, energy consumption, natural resource endowments, environmental regulation, foreign direct investment, economic development level (27, 42–44), and other factors on EE.

The research on both urbanization and EE mainly focuses on their spatiotemporal coupling characteristics (45, 46) and the impact of urban construction on EE (44). The unit of analysis of most studies is typically a whole country, urban agglomerations, or provinces. For example, at the scale of the whole country, Liu et al. (6, 45) analyzed the comprehensive urbanization level, energy EE, and their coupling coordination degree in 281 prefecture-level cities in China over the past 11 years. At the scale of urban agglomerations, Zhou et al. (47) focused on the impact of urbanization level and population urbanization lags on EE in the Bohai-rim urban agglomeration. At the province level, Yao et al. (44) used the spatial Durbin model to explore the multi-dimensional impact mechanism of urbanization in 30 provinces in China from 2008 to 2017, along with the internal structural effect of each dimension on EE. However, these studies have paid little attention to the relationship between the UPLSPs and EE.

**Figure 1:**
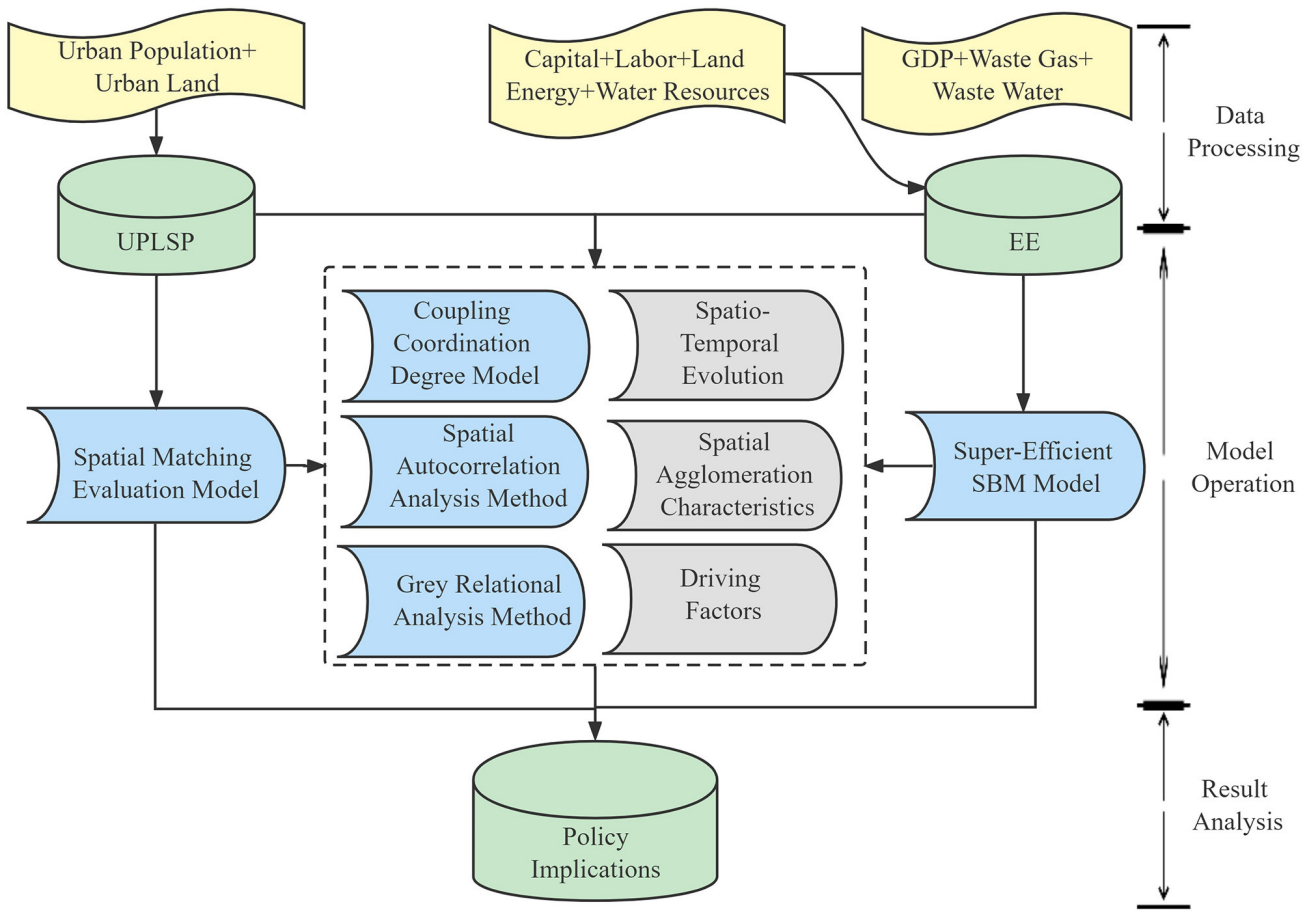
Research framework.

Therefore, taking Hubei Province as the research area, this study first calculates the UPLSPs, EE, and their coupling coordination degree for 12 cities in Hubei Province from 2000 to 2019 by constructing a spatial matching evaluation model, super-efficiency SBM model, and coupling coordination degree model. Then, spatial autocorrelation and gray relational analysis methods are used to explore the spatiotemporal evolution and driving factors of the coupling coordination degree between the UPLSPs and EE. Finally, on this basis, policy recommendations are made for new urbanization development and ecological civilization construction in Hubei Province. Further, this study provides a new framework for urban environmental assessment and urban planning decision-making. The research framework is shown in Figure 1.

## Study Area

**Figure 2:**
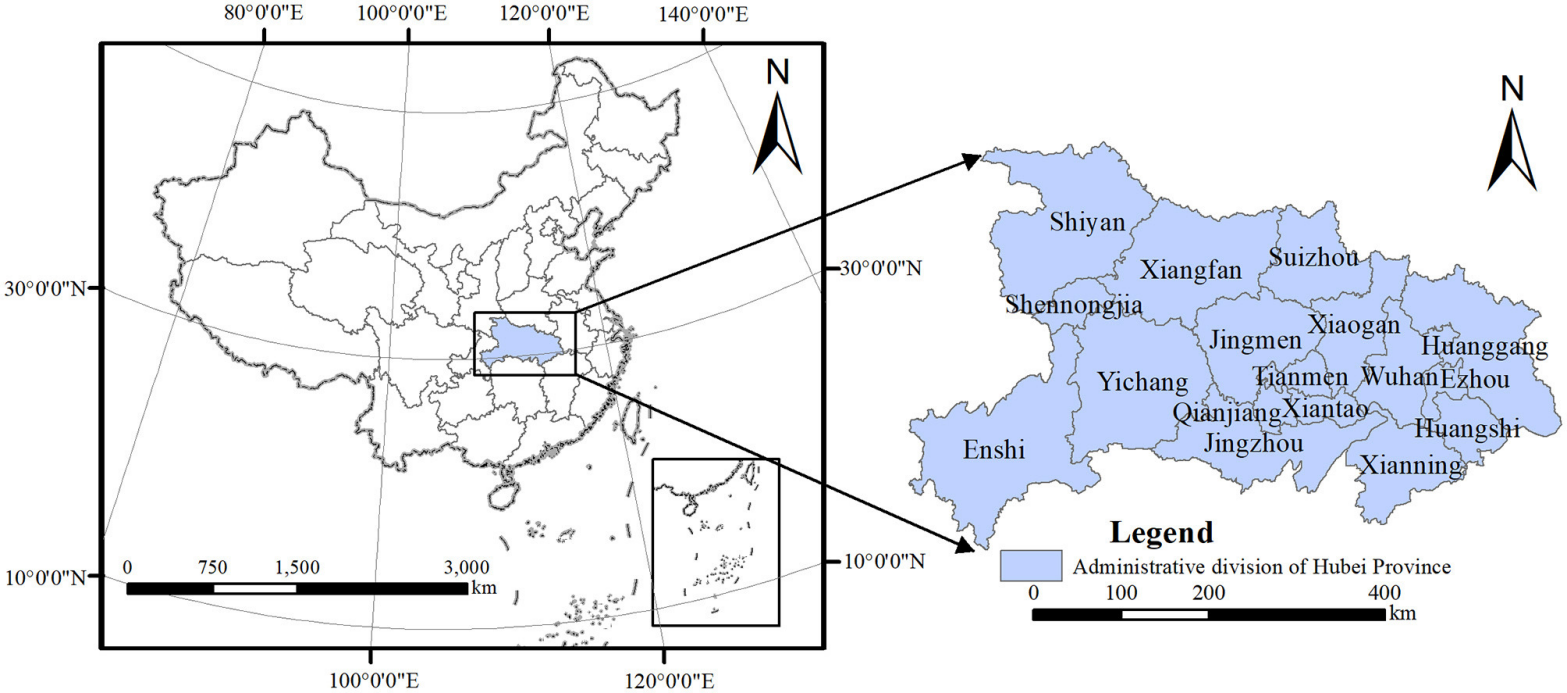
Study area (Source of base map: the open source map data service provided by the National Platform for Common GeoSpatial Information Services: https://www.tianditu.gov.cn).

Hubei Province is located in the middle of China (Figure 2) and spans east longitude 108°21'42”−116°07'50” and north latitude 29°01'53”−33°6'47”. It is about 740 kilometers from east to west and 470 kilometers from north to south. Hubei Province has 12 prefecture-level cities, three provincial municipalities, one autonomous prefecture, and one forest district. By the end of 2019, the permanent resident population of Hubei Province was 59.27 million, accounting for 4.23% of the national population. The total land area of the province was 185,900 square kilometers, accounting for 1.94% of the nation's total area (3).

Hubei Province has superior geographical conditions and is a powerful economic province in China. As an important part of the national development strategy of the Yangtze River Economic Belt and the rise of central China, it is necessary to actively promote the construction of new urbanization and ecological civilization. However, with the accelerated development of urbanization and industrialization, the contradiction between humans and land, economic development and environmental protection, in Hubei Province becomes more apparent. Therefore, this paper selects Hubei Province as the research area, and the findings have reference value for other cities. Moreover, due to the functional attributes of the city and the suitability of the research, this study considers 12 prefecture-level cities in Hubei Province.

## Data and Methods

### Data Sources and Processing

The data come from the *China Urban Construction Statistical Yearbook* (2000–2019), the *China City Statistical Yearbook* (2001–2020), and the *Hubei Statistical Yearbook* (2001–2020). Based on the data on adjacent years, the missing data for individual cities are reconstructed using the moving average and trend extrapolation methods.

The EE indicators constructed in this paper include input, desired output, and undesired output, as shown in Table 1. The input indicators include capital, labor, land, energy, and water resources; the desired output indicators are captured with regional gross domestic product (GDP) and deflating GDP, with 2000 identified as the baseline to eliminate the impact of price factors. The undesired output indicators include both waste gas and waste water. In addition, since the capital stock cannot be obtained directly from the statistical yearbook, this paper uses the capital stock, calculated through the continuous inventory method, as the capital investment; the formula is as follows:


(1) K_it=K_(it−1)(1−δ)+I_it

where *K* represents the capital stock, *i* represents the region, *t* represents the period, δ represents the depreciation rate, and *I* represents the total investment in fixed assets in the whole country. Following prior research (50), the depreciation rate is 9.6%. The total investment in fixed assets in the whole country in 2000 is divided by 10% as the initial capital stock *K_0*. According to the investment in fixed assets price index, the total investment in fixed assets in the whole country is converted into the constant price based on the year 2000.

**Table 1:** Evaluation index system of EE.

**Target level**	**Criterion level**	**Indicator level**	**Literature source**
Input	Capital	Total investment in fixed assets in the whole country/100 million yuan (Y1)	(25, 26, 33, 46, 48, 49)
	Labor	Number of employees at the end of the period/10,000 people (Y2)	(25, 26, 33, 46, 48, 49)
	Land	Built-up area/km^2^ (Y3)	(26, 33, 49)
	Energy	Annual electricity consumption/100 million kW.h (Y4)	(33, 48, 49)
	Water resources	Total water resources/100 million cu.m^3^ (Y5)	(26, 27, 48, 49)
Desired output	Economic growth	GDP/100 million yuan (Y6)	(25–27, 46, 48, 49)
Undesired output	Waste water	Industrial wastewater discharge/10,000 tons (Y7)	(26, 27, 33, 46, 48, 49)
	Waste gas	Industrial SO_2_ emissions/ton (Y8)	(27, 33, 48, 49)
		Industrial smoke (powder) dust emissions/ton (Y9)	(26, 27, 33, 46, 48, 49)

In the multi-index evaluation system, each evaluation indicator usually has different dimensions and orders of magnitude, owing to its different attributes. Therefore, this paper uses the min-max normalization method to standardize the original data (51, 52), as follows:


(2) $$\begin{array}{r} \text{positive indicators}:\;{\text{Z}}_{\text{ij}}=\frac{X_{ij}-min\left(X_{ij}\right)}{max\left(X_{ij}\right)-min\left(X_{ij}\right)} \end{array}$$

(3) $$\begin{array}{r} \text{negative indicators}:\;{\text{Z}}_{\text{ij}}=\frac{max\left(X_{ij}\right)-X_{ij}}{max\left(X_{ij}\right)-min\left(X_{ij}\right)} \end{array}$$

where *Z*_*ij*_ is the standard value of the index *j* in the *i*^th^ year, *X*_*ij*_ is the original data of the index *j* in the *i*^th^ year, and max(*X*_*ij*_) and min(*X*_*ij*_) are the maximum and minimum values of the *j*^th^ index, respectively.

### Methods

#### Spatial Matching Evaluation Model

Referring to the research on spatial matching and spatial agglomeration (53), this paper constructs the urban population–land spatial matching evaluation model, and uses the spatial density of the urban population and land to describe the coordinated relationship between people and land in the urbanization system. The model is as follows:


(4) $$\begin{array}{r} {Match}_{i}=\left(Pop_{i}/\sum_{i=1}^{n}Pop_{i}\right)\times {\left(Land_{i}/\overset{n}{\underset{i=1}{\sum }}Land_{i}\right)}^{-1}-1 \end{array}$$

where *Match*_*i*_ is the urban population–land spatial mismatching degree in the *i*^th^ area, and *Pop*_*i*_ and *Land*_*i*_ are the evaluation values for the urban population and urban land in the *i*^th^ area. The urban population and land measures are constructed using urban population size data and data on the built-up area, respectively; all data are obtained from the *China Urban Construction Statistical Yearbook*.

The absolute value of *Match*_*i*_ illustrates the urban population–land spatial matching degree, and *abs*(*Match*_*i*_)∈[0, +∞). When the value of *abs*(*Match*_*i*_) gradually increases, the urban population–land spatial matching degree tends to weaken; this means that the urban population and land in this region are uncoordinated in the spatial dimension. In contrast, when *abs*(*Match*_*i*_) gradually decreases, the urban population–land spatial matching degree tends to increase; this indicates that the urban population and land in this region are coordinated.

According to the sign on *Match*_*i*_, the types of UPLSPs can be divided into “urban population concentration,” “urban land concentration,” and “urban population–land relative matching.” Urban population concentration indicates that the urban population is more concentrated than the land in this area, while urban land concentration suggests the reverse. Finally, urban population–land relative matching indicates that the spatial distribution of the two components is balanced. To judge the strength of the urban population–land spatial matching degree, the grades of UPLSPs are divided into “relative matching,” “low mismatch,” “moderate mismatch,” and “high mismatch,” as shown in Table 2.

**Table 2:** Types and grades of UPLSPs.

**UPLSPs type**	**UPLSPs grade**	**Value range**	**UPLSPs type**	**UPLSPs grade**	**Value range**
Urban land concentration	High mismatch	*Match* < −0.5	Urban population concentration	Low mismatch	0.1 ≤ *Match* <0.3
Urban land concentration	Moderate mismatch	−0.5 ≤ *Match* < −0.3	Urban population concentration	Moderate mismatch	0.3 ≤ *Match* <0.5
Urban land concentration	Low mismatch	−0.3 ≤ *Match* < −0.1	Urban population concentration	High mismatch	*Match* ≥ 0.5
Urban population–land relative matching	Relative matching	−0.1 ≤ *Match* <0.1			

Based on the above urban population–land spatial matching evaluation model, the regional comprehensive mismatching degree evaluation model is defined as:


(5) $$Zmatch={\left[\frac{\left(\sum_{i=1}^{n}Match_{i}{}^{2}\right)}{n}\right]}^{\frac{1}{2}}$$

where *Zmatch* is the urban population–land comprehensive mismatching degree of the upper-level region, and *Match*_*i*_ is the same for the lower-level region. *Zmatch* is a comprehensive measure of the degree of urban population–land mismatching in the upper-level region containing the lower-level region. For *Zmatchϵ* [0, +∞, the larger the *Zmatch* value, the higher the comprehensive mismatch of the upper region, and the more unbalanced the spatial distribution of the urban population and land in this region.

#### Super-Efficient SBM Model

The common EE measurement method is the DEA model. However, traditional DEA models are mostly radial or angular and do not consider the problem of factor relaxation. Therefore, Tone proposed a non-radial and non-angular SBM model (54, 55), which considered not only the proportional improvement between the current state of the invalid DMU and the strong target value, but also the relaxation improvement. However, it is still a problem that the effective units cannot be further sorted and distinguished. Therefore, this study adopts the super-efficiency SBM model; that is, the SBM model is extended by considering the changes in production technology that occurred each year during the study period (56). The efficiency is estimated in a non-ray manner. Equation (6) shows this expression:


(6) $$\rho =\min\frac{1-\frac{1}{m}\sum_{i=1}^{m}\frac{s_{h}^{-}}{x_{hk}}}{1+\frac{1}{q_{1}+q_{2}}\left(\sum_{r=1}^{q_{1}}\frac{s_{r}^{-}}{y_{rk}}+\sum_{t=1}^{q_{2}}\frac{s_{t}^{b-}}{y_{hk}^{b}}\right)}$$

$$\begin{array}{l} s.t.\overset{n}{\underset{j=1,j\neq k}{\sum }}x_{h1}\lambda_{1}-s_{h}^{-}\leq x_{ik};\overset{n}{\underset{l=1,l\neq k}{\sum }}y_{hl}^{b}\lambda_{1}-s_{r}^{b-}\leq y_{rk} \end{array}$$

$$\begin{array}{l} 1+\frac{1}{q_{1}+q_{2}}\left(\overset{q_{1}}{\underset{r=1}{\sum }}\frac{s_{r}^{-}}{y_{rk}}+\overset{q_{2}}{\underset{t=1}{\sum }}\frac{s_{t}^{b-}}{y_{hk}^{b}}\right)>\;0 \end{array}$$

$$\begin{array}{l} \lambda_{h},s_{h}^{-},s_{r}^{+}\geq \;0 \end{array}$$

where ρ denotes EE; *x*_*hk*_, *y*_*rk*_, and $y_{hk}^{b}$ denote the *h*^th^ input factor, the *r*^th^ desired output, and the *h*^th^ undesired output of the *k*^th^ prefecture-level city, respectively; $s_{h}^{-}$, $s_{r}^{-}$, and $s_{t}^{b-}$ denote slack variables of the input factors, desired output, and undesired output, respectively; λ_1_ denotes the constraint condition; and *h* = 1, 2, …, *q*_1_; *t* = 1, 2, …, *q*_2_; and *l* = 1, 2, …, n(*j*≠*k*).

#### Coupling Coordination Degree Model

Coupling coordination degree refers to the degree of benign coupling in the interaction relationship; it can be used to characterize whether the functions promote each other at a high level or restrict each other at a low level (57, 58). This study uses it to measure the coordinated relationship between the UPLSPs and EE.

##### Coupling Degree Model

Based on the relevant research results (59, 60), the coupling degree model of UPLSPs and EE is constructed as follows:


(7) $$\begin{array}{r} C={\left[\frac{f\left(x\right)\times g\left(y\right)}{{\left(\frac{f\left(x\right)+g\left(y\right)}{2}\right)}^{2}}\right]}^{\frac{1}{2}} \end{array}$$

where *C* is the coupling degree between UPLSPs and EE, and *C*∈[0, 1]. When the value of *C* is smaller, the mutual interaction between UPLSPs and EE is lower, and vice versa. When *C* = 1, the optimal coordination state of the two is achieved. The functions *f*(*x*) and *g*(*y*) are UPLSPs and EE, respectively.

##### Coupling Coordination Degree Model

The coupling coordination index is introduced to construct the coupling coordination degree model of UPLSPs and EE, and the model is as follows (61, 62):


(8) $$\begin{array}{r} D=\sqrt{C\times T} \end{array}$$

(9) $$\begin{array}{r} T=\alpha f\left(x\right)+\beta g\left(y\right) \end{array}$$

where *D* is the coupling coordination degree of UPLSPs and EE, *C* is the coupling degree of UPLSPs and EE, *T* is the comprehensive coordination index of UPLSPs and EE, and α and β are the contributions of UPLSPs and EE to urban development, respectively. Considering the same contribution, α = β = 0.5.

As in previous studies (63, 64), the coupling coordination degree of UPLSPs and EE is divided into three intervals (imbalanced recession interval, transitional harmonic interval, and coordinated development interval) and ten types, as shown in Table 3.

**Table 3:** Classification of the coupling coordination degree.

***D***	**Coupling coordination type**	**Coupling coordination interval**	***D***	**Coupling coordination type**	**Coupling coordination interval**
0 ≤ D <0.1	Extremely imbalanced	Imbalanced recession interval	0.5 ≤ D <0.6	Primary coordination	Coordinated development interval
0.1 ≤ D <0.2	Moderately imbalanced		0.6 ≤ D <0.7	Intermediate coordination	
0.2 ≤ D <0.3	slightly imbalanced		0.7 ≤ D <0.8	good coordination	
0.3 ≤ D <0.4	barely harmonic coordination	Transitional harmonic interval	0.8 ≤ D <0.9	high-quality coordination	
0.4 ≤ D <0.5	harmonic coordination		0.9 ≤ D ≤ 1	extreme coordination	

#### Spatial Autocorrelation Analysis Model

Spatial autocorrelation is an important indicator that tests whether the attribute value of a certain element is significantly associated with the attribute value of its adjacent spatial points. It is divided into global spatial autocorrelation and local spatial autocorrelation (65, 66). Global spatial autocorrelation is used to measure whether there is agglomeration or dispersion in the spatial distribution of element attribute values. It is usually expressed by the global Moran's I index, the calculation of which is as follows (15, 44, 67):


(10) $$I=\frac{\sum_{i=1}^{n}\sum_{j=1}^{n}W_{ij}\left(x_{i}-x\right)\left(x_{j}-x\right)}{S^{2}\sum_{i=1}^{n}\sum_{j=1}^{n}W_{ij}}$$

where *I* is the global Moran's I index; *n* is the number of prefecture-level cities; *x*_*i*_ and *x*_*j*_ are the coupling coordination degrees of UPLSPs and EE in regions *i* and *j*, respectively; $\overset{\bar{}}{x}$ is the average value of the coupling coordination degree of UPLSPs and EE; *S*^2^ is the sample variance; and $S^{2}=\frac{1}{n}\sum_{i=1}^{n}{\left(x_{i}-x\right)}^{2}$. *W*_*ij*_is the spatial weight matrix of regions *i* and *j* (when regions *i* and *j* are adjacent, *W*_*ij*_ =1; when regions *i* and *j* are not adjacent, *W*_*ij* =_0). The value of Moran's I index is in the range of [−1,1]. Moran's I index values greater than zero indicate a positive spatial correlation, less than zero indicate a negative spatial correlation, and equal to zero indicate no spatial correlation.

Local spatial autocorrelation is used to measure the correlation of each spatial element attribute in the local position. It is usually expressed by the local Moran's I index, the calculation of which is as follows (68):


(11) $$\begin{array}{r} I_{i}=\frac{\left(x_{i}-\bar{x}\right)}{S^{2}}\overset{n}{\underset{j=1}{\sum }}W_{ij}\left(x_{j}-\bar{x}\right) \end{array}$$

where *I*_*i*_ is the local Moran's I index; the meanings of *n, x*_*i*_, *x*_*j*_, $\bar{x}$, *S*^2^, and *W*_*ij*_are shown above. A value of *I*_*i*_ >0 indicates the spatial aggregation of similar values (high–high or low–low) around the unit in the area, and a value <0 indicates the spatial aggregation of dissimilar values (high–low or low–high).

#### Gray Relational Analysis Method

Gray system theory is a method proposed by Deng (69) in the 1980's to study the uncertainty problem with little data and poor information. Gray relational analysis is a quantitative method used to judge whether the corresponding sequences are closely related according to the geometric similarity of sequence curves, which can measure the degree of association between factors. The calculation steps are as follows (70, 71):

1. Determine the reference sequence and comparison sequence, and use the mean normalization method for data standardization. This study selects the coupling coordination degree of UPLSPs and EE in Hubei Province as the reference sequence, denoted as *X*_*O*_(*k*), and the driving factors as the comparison sequence, denoted as *X*_*i*_(*k*). The normalized reference sequence and comparison sequence are ${X_{O}}^{{}^{\prime }}\left(k\right)$ and ${X_{i}}^{{}^{\prime }}\left(k\right)\left(i=1,2,3,\;\ldots ,n\;\right)$.
2. Calculate the relational coefficient ζ_*i*_(*k*).
  (12) $$\begin{array}{r} \zeta_{i}\left(k\right)\\ =\frac{\underset{i}{\min}\underset{k}{\min}|{X_{O}}^{{}^{\prime }}\left(k\right)-{X_{i}}^{{}^{\prime }}\left(k\right)|+\rho \underset{i}{\max}\underset{k}{\max}|{X_{O}}^{{}^{\prime }}\left(k\right)-{X_{i}}^{{}^{\prime }}\left(k\right)|}{\left|{X_{O}}^{{}^{\prime }}\left(k\right)-{X_{i}}^{{}^{\prime }}\left(k\right)|+\rho \underset{i}{\max}\underset{k}{\max}|{X_{O}}^{{}^{\prime }}\left(k\right)-{X_{i}}^{{}^{\prime }}\left(k\right)\right|} \end{array}$$
  $$\begin{array}{l} {X_{i}}^{{}^{\prime }}\left(k\right)=\frac{X_{i}\left(k\right)}{{\bar{X}}_{i}\left(k\right)}{\bar{X}}_{i}\left(k\right)=\frac{1}{n}\overset{n}{\underset{k=1}{\sum }}X_{i}\left(k\;\right) \end{array}$$
  where $\left|X_{O}^{\prime }(k)-X_{i}^{\prime }(k)\right|$ is the minimum absolute value of two levels of two sequences, $\left|X_{O}^{\prime }(k)-X_{i}^{\prime }(k)\right|$ is the maximum absolute value of two levels of two sequences; ρ is the resolution coefficient; and ρ = 0.5.
3. Calculate the relational degree *r*_*i*_:
  (13) $$\begin{array}{r} r_{i}=\frac{1}{n}\overset{N}{\underset{k=1}{\sum }}\zeta_{i}\left(k\right),i=1,2,3,\;\ldots ,n \end{array}$$

The value range of the relational degree *r*_*i*_ is [0, 1]; the larger the value of *r*_*i*_, the greater the correlation between the indicators and the stronger the coupling. In addition, as in the literature (72, 73), the relational degree of the coupling coordination degree of UPLSPs and EE is divided into six types, as shown in Table 4.

**Table 4:** Classification of the relational degree.

**Grade**	**No correlation**	**Low correlation**	**Moderate correlation**	**High** **correlation**	**Strong correlation**	**Complete** **correlation**
*r_*i*_*	0	(0, 0.35)	(0.35, 0.65)	(0.65, 0.85)	(0.85, 1)	1

## Results

### Spatiotemporal Evolution of UPLSPs in Hubei Province

This study uses cross-sectional data from 2000, 2005, 2010, 2015, and 2019 to analyze the UPLSPs of Hubei Province along the time and space dimensions based on the spatial matching evaluation model. The results are shown in Table 5. The comprehensive mismatching degree of Hubei Province shows an overall downward trend, from 0.3228 in 2000 to 0.2530 in 2019, and the type of UPLSPs is always urban population concentration. This indicates that the level of urban population–land spatial matching has been gradually increasing. The main reason for this is because, with the development of the social economy and the improvement of living standards, more people have moved from rural areas to cities, resulting in an increase of the urban population and population density.

**Table 5:** UPLSPs in Hubei Province from 2000 to 2019.

**Number**	**City**	**2000**	**2005**	**2010**	**2015**	**2019**
1	Wuhan	0.4270	0.4288	0.2169	0.1027	0.1224
2	Huangshi	−0.0190	−0.2064	0.1287	0.4855	0.1354
3	Shiyan	−0.3792	−0.3557	−0.1882	−0.1431	−0.2460
4	Yichang	−0.1046	−0.1954	−0.1805	−0.2847	−0.3411
5	Xiangyang	−0.1523	−0.1304	−0.1276	−0.1332	−0.0652
6	Ezhou	−0.4224	−0.3897	−0.2073	−0.1453	−0.3260
7	Jingmen	−0.4628	−0.3417	−0.2068	−0.0389	0.0692
8	Xiaogan	−0.4170	−0.5412	−0.3374	0.2901	0.4528
9	Jingzhou	−0.0011	0.2730	0.0777	0.1243	0.3045
10	Huanggang	−0.1921	−0.0221	−0.0415	−0.2662	−0.1050
11	Xianning	−0.3107	−0.4261	−0.5044	−0.2696	−0.2683
12	Suizhou	0.4356	0.7369	−0.2239	0.1478	−0.2484
Comprehensive mismatching degree of Hubei Province		0.3228	0.3840	0.2345	0.2333	0.2530

At the city level, the urban population–land spatial matching degree in Hubei Province from 2000 to 2019 increased in Wuhan, Shiyan, Xiangyang, Ezhou, Jingmen, Huanggang, Xianning, and Suizhou. It decreased in Huangshi, Yichang, Xiaogan, and Jingzhou. In 2000, Shiyan, Yichang, Xiangyang, Ezhou, Jingmen, Xiaogan, Huanggang, and Xianning were classified as urban land concentration, Wuhan and Suizhou were urban population concentration, and Huangshi and Jingzhou were urban population–land relative matching. In 2019, Shiyan, Yichang, Ezhou, Huanggang, Xianning, and Suizhou were urban land concentration; Wuhan, Huangshi, Xiaogan, and Jingzhou were urban population concentration; and Xiangyang and Jingmen were matched. The UPLSPs type in Hubei Province is mainly urban land concentration, and there has been a transformation from concentrations of urban land to urban populations.

**Figure 3:**
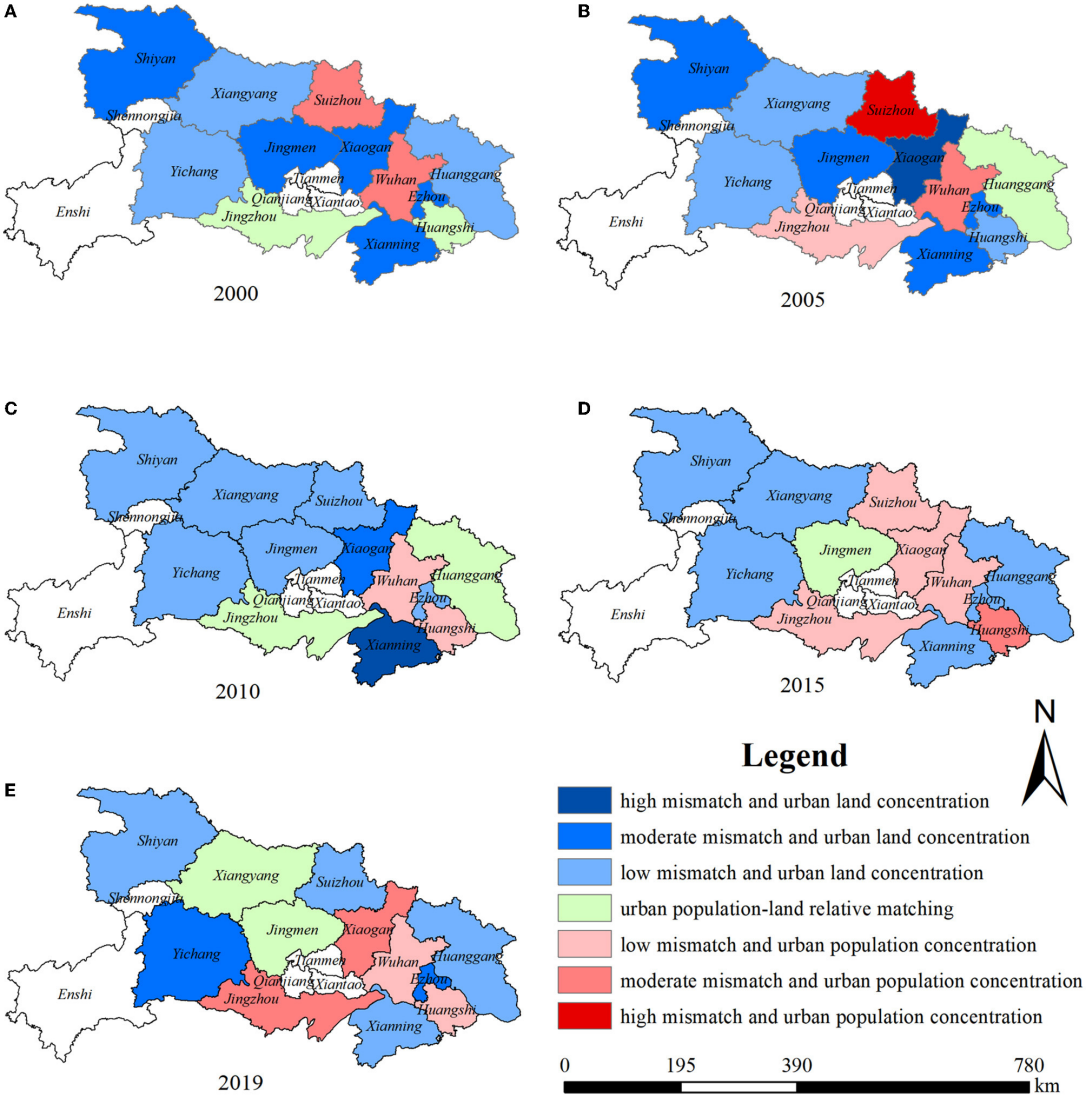
**(A–E)** Spatial distribution of UPLSPs in Hubei Province from 2000 to 2019 (Source of base map: the open source map data service provided by the National Platform for Common GeoSpatial Information Services: https://www.tianditu.gov.cn).

Figure 3 shows the evolution of the spatial pattern of UPLSPs. There were seven cities with a moderate mismatch in 2000, which reduced to four in 2019. Three cities has a low mismatch in 2000, which increased to six in 2019. The number of matched cities was relatively stable, with two in 2000, 2010, and 2019, and only one in other years. This indicates that urban population–land spatial matching has spatial heterogeneity. Moreover, the overall matching level is not high but shows an upward trend annually.

### Spatiotemporal Evolution of EE in Hubei Province

This study uses the super-efficiency SBM model and MATLAB software to calculate the EE of 12 cities in Hubei Province in 2000, 2005, 2010, 2015, and 2019. According to Table 6, the average value of EE in Hubei Province increased from 0.6459 in 2000 to 0.6618 in 2019, with an average annual growth rate of 0.1281%. The change is “N”-shaped—first rising, then decreasing, and then rising—and the overall level of EE is low, but the changes are not large.

**Table 6:** EE in Hubei Province from 2000 to 2019.

**Number**	**City**	**2000**	**2005**	**2010**	**2015**	**2019**	**Average value**	**Average annual growth rate (%)**
1	Wuhan	1.2294	1.3761	1.0453	1.1646	1.2163	1.2063	−0.0564
2	Huangshi	0.4425	0.5912	0.4595	0.5795	0.7781	0.5702	3.0152
3	Shiyan	0.4330	0.6057	0.4596	0.4653	0.5031	0.4933	0.7929
4	Yichang	0.3877	0.4024	0.2845	0.2717	0.3185	0.3330	−1.0295
5	Xiangyang	0.3703	0.4451	0.2847	0.2536	0.3571	0.3422	−0.1909
6	Ezhou	0.4581	0.6117	0.6084	1.0097	1.1325	0.7641	4.8789
7	Jingmen	1.0397	1.1617	1.0021	0.6944	1.1066	1.0009	0.3287
8	Xiaogan	0.4718	0.5897	0.3402	0.2285	0.3068	0.3874	−2.2396
9	Jingzhou	0.4216	0.5983	0.4032	0.4201	0.5149	0.4716	1.0577
10	Huanggang	0.3905	0.5382	0.1905	0.2056	0.2480	0.3146	−2.3611
11	Xianning	0.3954	0.5413	0.2466	0.2597	0.2799	0.3446	−1.8018
12	Suizhou	1.7104	1.4495	1.4708	1.2866	1.1801	1.4195	−1.9344
	Hubei	0.6459	0.7426	0.5663	0.5699	0.6618	0.6373	0.1281

At the city level, the EE values for Huangshi, Shiyan, Ezhou, Jingmen, and Jingzhou increased slightly from 2000 to 2019, and the average annual growth rates of Huangshi and Ezhou were higher, at 3.0152% and 4.8789%, respectively. The EE values of Wuhan, Yichang, Xiangyang, Xiaogan, Huanggang, Xianning, and Suizhou decreased from 2000 to 2019, and the average annual growth rates of Xiaogan, Huanggang, Suizhou, and Xianning were higher: −2.2396%, −2.3611%, −1.9344%, and −1.8018%, respectively.

**Figure 4:**
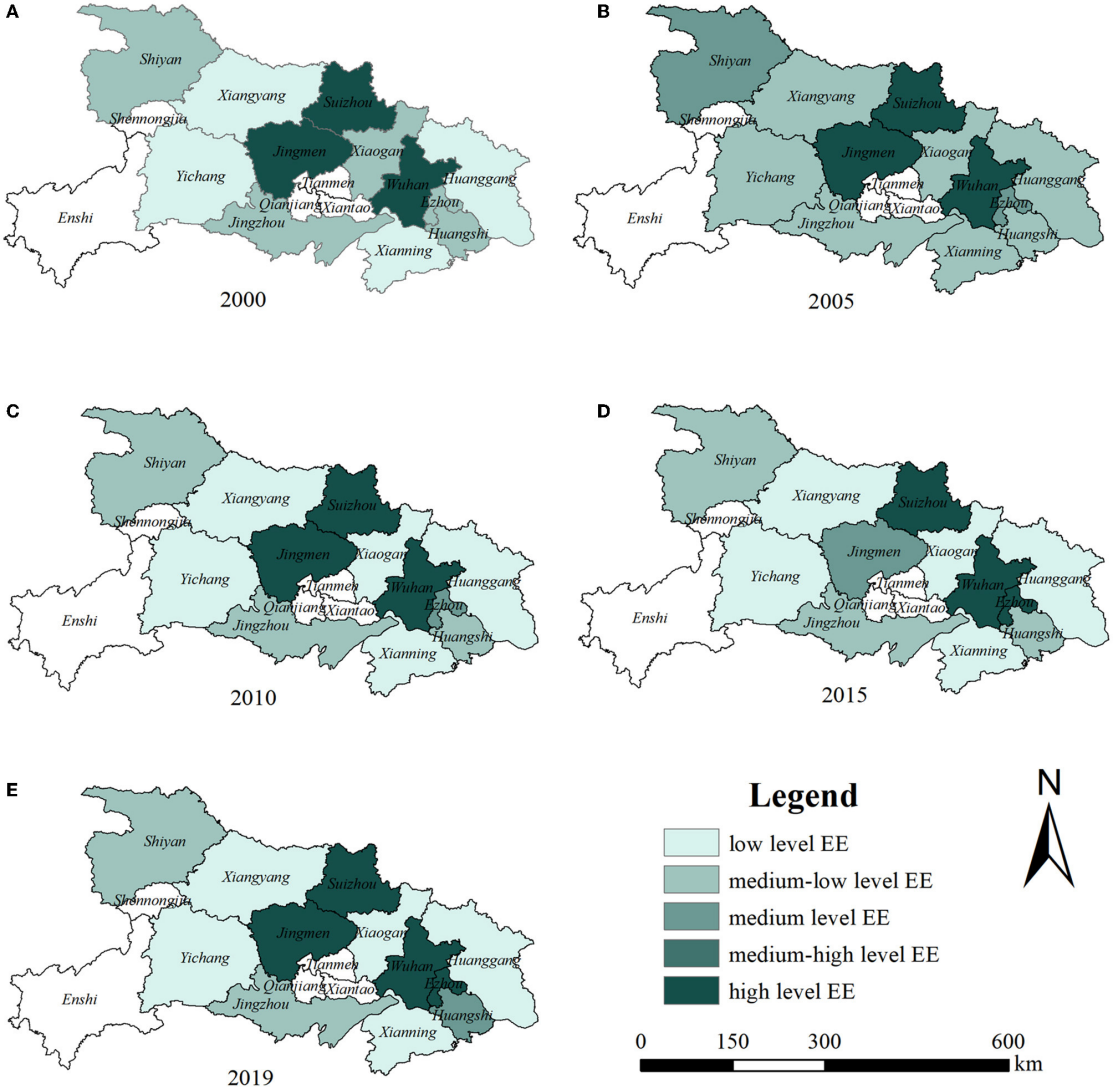
**(A–E)** Spatial distribution of EE in Hubei Province from 2000 to 2019 (Source of base map: the open source map data service provided by the National Platform for Common GeoSpatial Information Services: https://www.tianditu.gov.cn).

Subsequently, using the natural breaks method, the EE value of Hubei Province is divided into five types: 0–0.4 is low, 0.4–0.6 is medium-low, 0.6–0.8 is medium, 0.8–1.0 is medium-high, and 1.0–2.0 is high. Figure 4 depicts the results, and the level of EE shows a gradual upward trend. There were three cities with a high level of EE in 2000; this increased to four by 2019. No city was included in the medium-high group in 2000, but this increased to one in 2019. Five cities were at the medium-low level of EE, which reduced to two by 2019. A the low level of EE, there were four cities in 2000 and five by 2019. In addition, there are obvious regional differences in EE across Hubei Province, with high levels found in the center of the region and low levels around the periphery. In 2000, only Wuhan, Jingmen, and Suizhou in central Hubei Province had high level of EE. In 2019, Wuhan, Ezhou, Jingmen, and Suizhou were coded high, and the EE levels of other cities were low, with a radial distribution from the center to the surrounding areas. The main reason for this may be that the cities in the central plain have a higher level of economic development, better transportation options, and higher levels of technological innovation, which promote the improvement of EE.

### Spatiotemporal Evolution of the Coupling Coordination Degree Between UPLSPs and EE in Hubei Province

**Figure 5:**
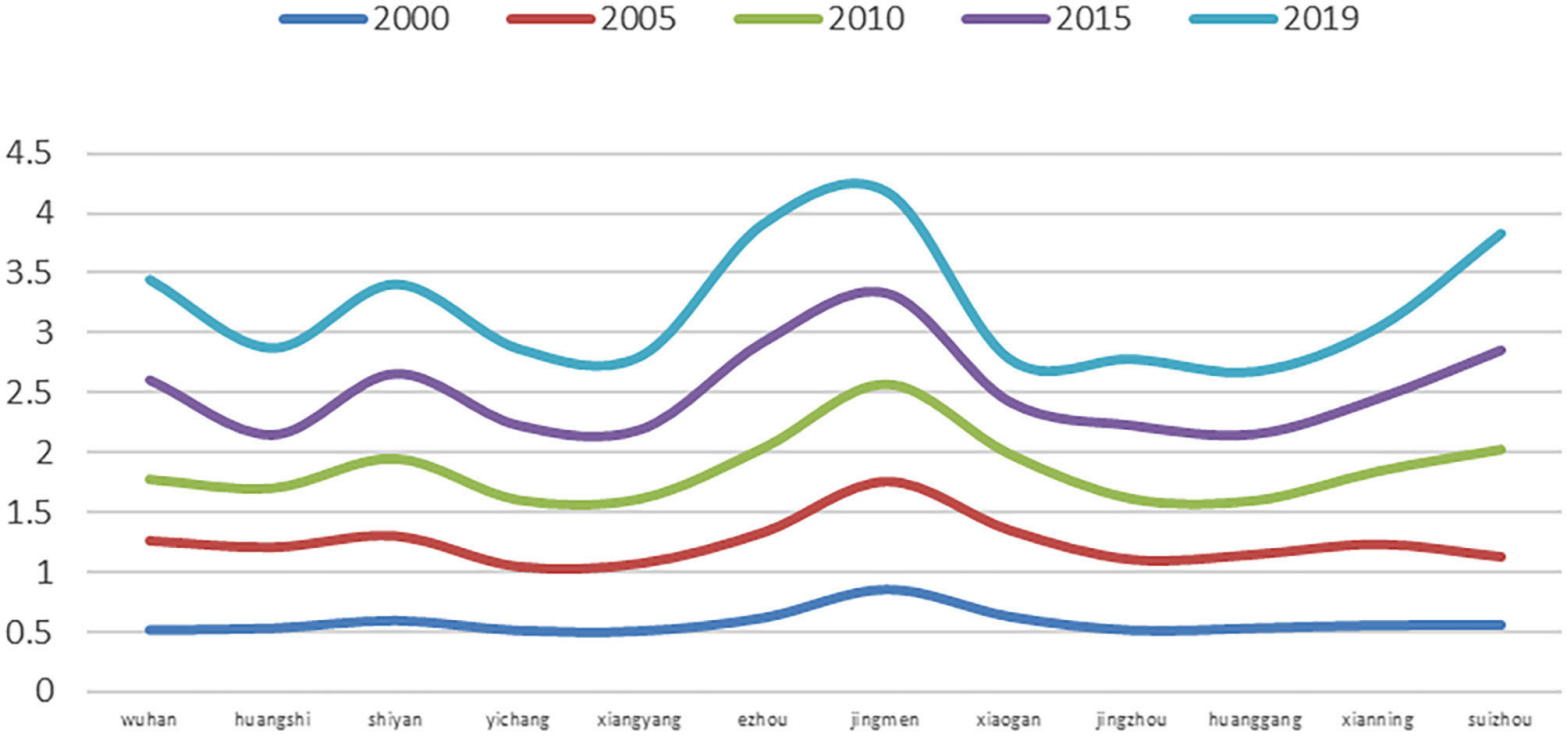
The coupling coordination degree between UPLSPs and EE in Hubei Province.

The results obtained using the coupling coordination degree model are shown in Table 7 and Figure 5. Based on Table 3, the relationship between UPLSPs and EE is classified into conflict and coordination. If the coupling coordination degree is <0.7, the relationship is considered conflictual; if it is >0.7, the relationship is considered one of coordination.

**Table 7:** The coupling coordination degree between UPLSPs and EE in Hubei Province from 2000-2019.

**Number**	**City**	**2000**	**2005**	**2010**	**2015**	**2019**	**Average value**	**Average annual growth rate (%)**
1	Wuhan	0.5208	0.7382	0.5145	0.8374	0.8300	0.6882	2.4836
2	Huangshi	0.5359	0.6691	0.4964	0.4503	0.7225	0.5748	1.5847
3	Shiyan	0.6007	0.6991	0.6469	0.7167	0.7406	0.6808	1.1078
4	Yichang	0.5173	0.5244	0.5609	0.6275	0.6378	0.5736	1.1081
5	Xiangyang	0.5123	0.5583	0.5448	0.5826	0.6100	0.5616	0.9224
6	Ezhou	0.6249	0.7071	0.7056	0.8959	0.9757	0.7818	2.3725
7	Jingmen	0.8610	0.8968	0.8058	0.7753	0.8325	0.8343	−0.1771
8	Xiaogan	0.6329	0.7147	0.6349	0.4446	0.3527	0.5560	−3.0311
9	Jinzhou	0.5185	0.5817	0.5112	0.6175	0.5527	0.5563	0.3362
10	Huanggang	0.5364	0.6089	0.4533	0.5593	0.5202	0.5356	−0.1612
11	Xianning	0.5610	0.6701	0.6111	0.6144	0.5873	0.6088	0.2412
12	Suizhou	0.5623	0.5623	0.8979	0.8386	0.9643	0.7651	2.8793
	Hubei Province	0.5820	0.6609	0.6153	0.6633	0.6938	0.6431	0.9294

The average coupling coordination degree of UPLSPs and EE in Hubei Province from 2000 to 2019 exhibits a slow upward trend, increasing from 0.5820 in 2000 to 0.6938 in 2019. This translates to a shift from primary coordination to intermediate coordination, with an average annual growth rate of 0.9294%. In addition, there is a conflictual relationship between UPLSPs and EE in Hubei Province, and the population–land spatial matching degree is lower than the EE. Therefore, the UPLSPs of Hubei Province restricted the development of EE during this period.

At the city level, the coupling coordination degree of UPLSPs and EE shows an overall upward trend from 2000 to 2019, including in Wuhan, Huangshi, Shiyan, Yichang, Xiangyang, Ezhou, Jingzhou, Xianning, and Suizhou. The areas with a downward trend include Jingmen, Xiaogan, and Huanggang. From 2000 to 2019, Jingmen had the highest average value of coupling coordination (0.8343), which is considered high-quality coordination; Huanggang had the lowest average value (0.5356), which is considered primary coordination. In 2009, only Jingmen had a coordination relationship, while the other cities were classified as exhibiting conflict, and the population–land spatial matching degrees were all lower than the EE values. Clearly, the UPLSPs in regions other than Jingmen constrained the development of EE. In 2019, the UPLSPs and EE of Wuhan, Huangshi, Shiyan, Ezhou, Jingmen, and Suizhou featured coordination relationships, while Yichang, Xiangyang, Xiaogan, Jingzhou, Huanggang, and Xianning exhibited conflictual relationships. The population–land spatial matching degree of Xiaogan was higher than the EE value, indicating that EE in this area restricted the development of UPLSPs. The patterns in the other cities were the opposite.

**Figure 6:**
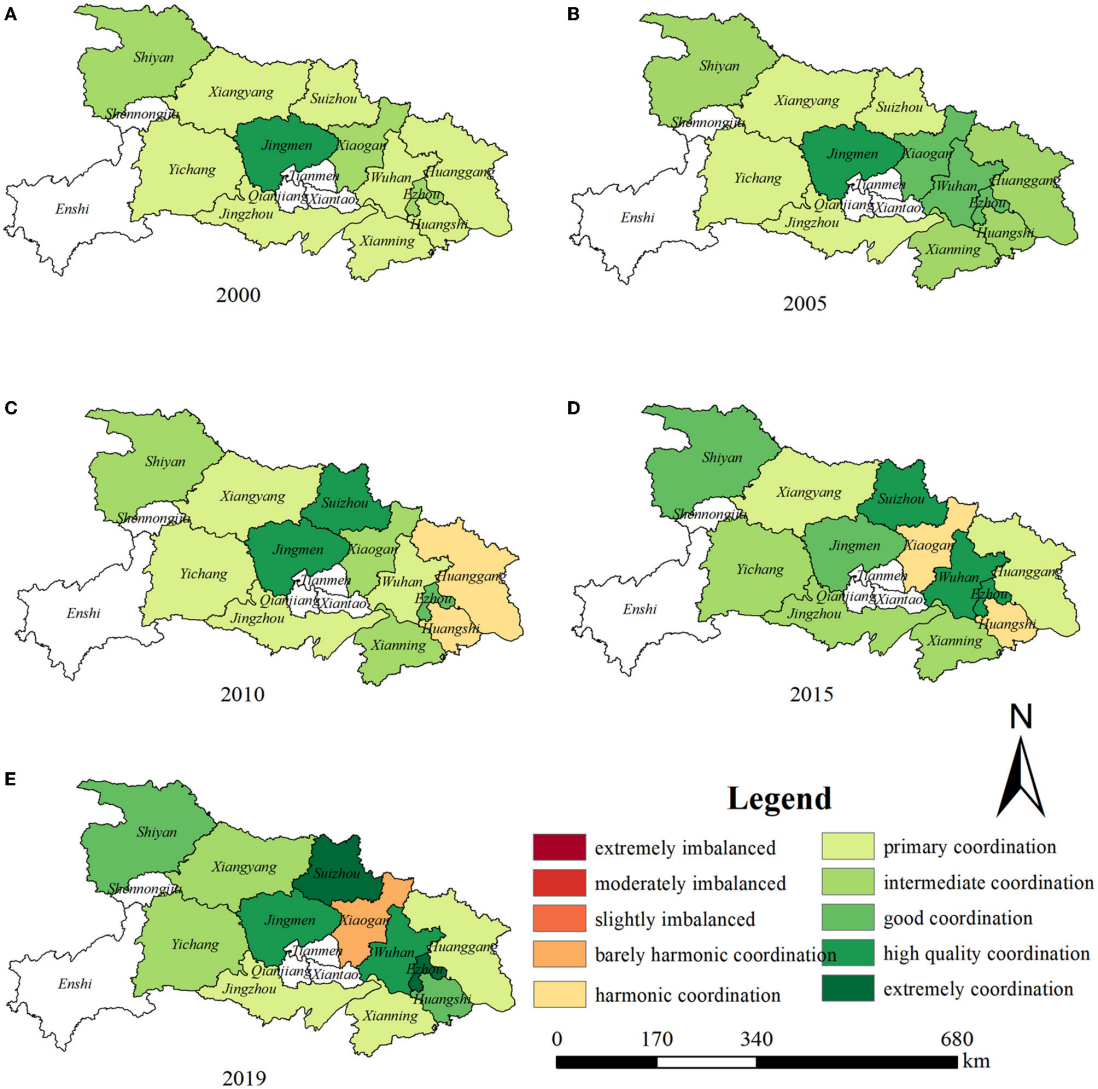
**(A–E)** Spatial distribution of the coupling coordination degree between UPLSPs and EE (Source of base map: the open source map data service provided by the National Platform for Common GeoSpatial Information Services: https://www.tianditu.gov.cn).

Figure 6 indicates that from 2000 to 2019, the coupling coordination degree of UPLSPs and EE in Hubei Province has improved: in 2000, there were eight cities with primary coordination, three with intermediate coordination, and one with high-quality coordination. In 2019, once city had barely harmonic coordination, three had primary coordination, two had intermediate coordination, two had good coordination, two had high-quality coordination, and two had extreme coordination. The spatial distribution of the coupling coordination degree of UPLSPs and EE is similar to that of EE; that is, higher values are found in the middle, with lower values found around the periphery. In 2000, Jingmen, in the central region of Hubei Province, had the highest coupling coordination degree, which involved high-quality coordination. By 2019, Suizhou and Ezhou featured extreme coordination, and Wuhan and Jingmen were coded as high-quality coordination. The main reason for this may be that the spatial distributions of population and land in these areas are relatively balanced, and the level of EE is high. UPLSPs and EE promote and influence each other, showing a high state of coupling.

Using Geoda software, the spatial autocorrelation analysis of the coupling coordination degree of UPLSPs and EE in Hubei Province is carried out using the geographical distance spatial weight matrix. The results are shown in Table 8. The global Moran's I index of the coupling coordination degree of each city was negative from 2000 to 2019. Except for 2015, the results are statistically significant at the 5% level, showing a significant negative spatial correlation. That is, the spatial distribution illustrates that cities with high coupling coordination are surrounded by cities with low coupling coordination, while cities with low coupling coordination are surrounded by those with high coupling coordination. Specifically, the global Moran's I index shows a trend of first decreasing, then increasing, and again decreasing, from −0.361 in 2000 to −0.438 in 2019. This reflects the gradual growth of spatial dispersion of coupling coordination degree across cities, as well as the gradual expansion of the difference in this degree.

**Table 8:** Moran's I index of the coupling coordination degree between UPLSPs and EE from 2000-2019.

	**2000**	**2005**	**2010**	**2015**	**2019**
Moran's I	−0.361	−0.462	−0.048	−0.425	−0.438
Z value	−1.9800	−2.0155	0.2677	−1.6054	−1.6991
*P* value	0.027**	0.014**	0.354	0.034**	0.033**

*** *p <0.01,*

** *p <0.05,*

* *p < 0.1*.

*The statistical inferences on the table are based on 999 random permutations proposed by Ansen Lin*.

The global Moran's I index only reveals the overall agglomeration type of the study area, and it is necessary to further identify the local spatial correlations and the spatial pattern distribution of the coupling coordination degree using a Moran scatterplot. The Moran scatterplot classifies the coupling coordination degree of UPLSPs and EE into four types, which fall in different quadrants. Quadrant I is the high–high agglomeration (H-H), indicating that the level of coupling coordination between the region and its surrounding areas is relatively high, and the degree of spatial difference between the two is small. Quadrant II is the low–high agglomeration (L-H), indicating that the region has a low level of coupling coordination, the surrounding areas are higher, and the degree of spatial difference between the two is relatively large. Quadrant III represents the low–low agglomeration (L-L), indicating that the level of coupling coordination between the region and the surrounding areas is low, and the spatial difference between the two is relatively small. Finally, Quadrant IV is the high–low agglomeration (H-L), indicating that the level of coupling coordination in the region is relatively high, while the surrounding areas are relatively low, and the degree of spatial difference between the two is relatively large.

**Figure 7:**
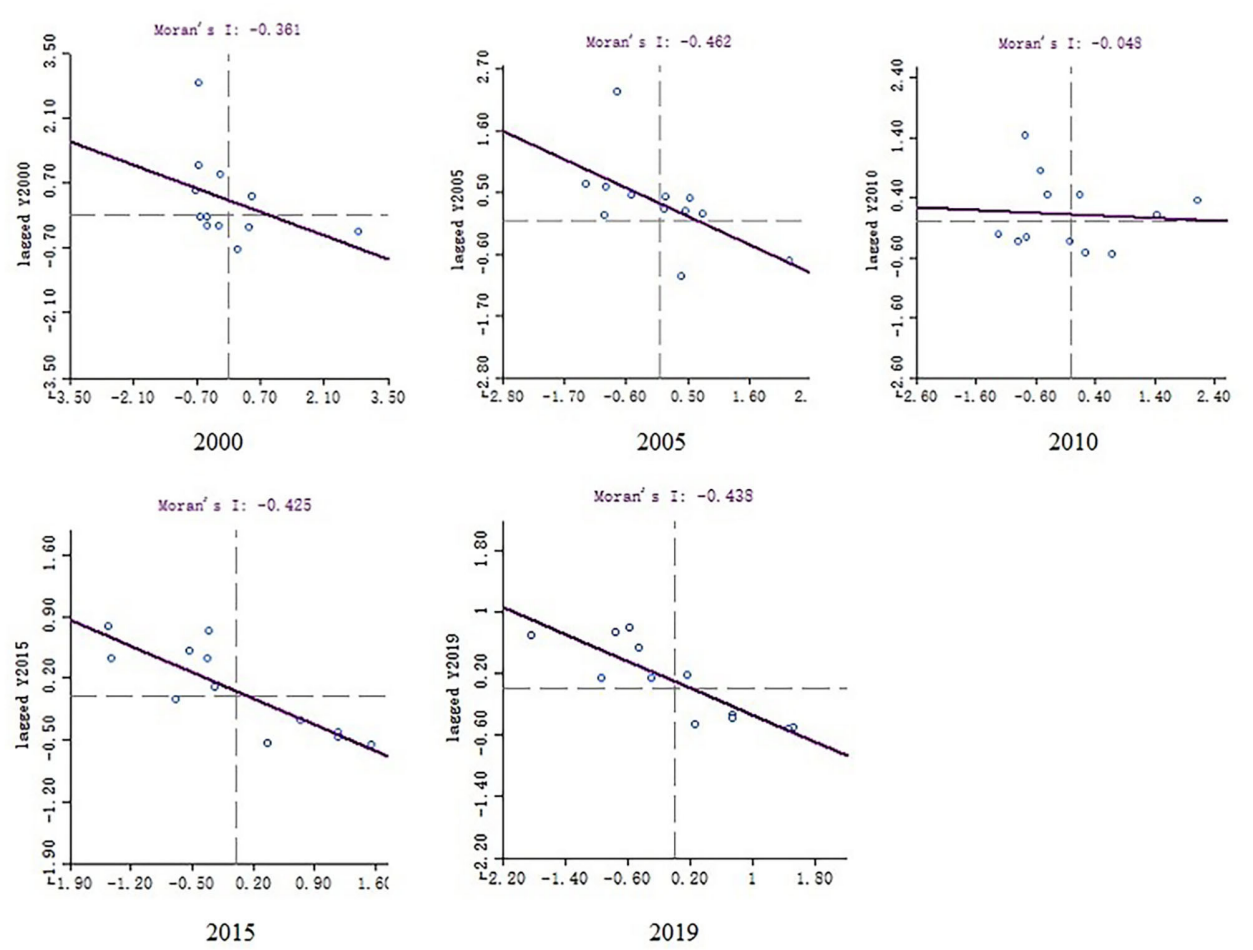
Moran scatterplot of the coupling coordination degree between UPLSPs and EE in Hubei Province from 2000-2019.

According to Figure 7 and Table 9, most cities in Hubei Province fell into Quadrants II and IV in 2000 and 2019. This suggests a significant negative spatial correlation between the coupling coordination degree of UPLSPs and EE, which has the characteristics of a discrete distribution; that is, the cites with high coupling coordination are adjacent to those with low coupling coordination. Cities in Quadrant II are L-H, indicating that the natural resource endowments of each city have strong heterogeneity and the gap in economic development is large, so the spatial connection is weak. Cities in Quadrant IV are H-L, indicating that these areas do not have a strong driving effect on the surrounding cities, and they even absorb the advantageous resources around them to vigorously develop themselves.

**Table 9:** Agglomeration pattern of the coupling coordination degree between UPLSPs and EE in Hubei Province.

	**H-H agglomeration**	**L-H agglomeration**	**L-L agglomeration**	**H-L** **agglomeration**
2000	Xiaogan	Jingzhou Yichang Xiangyang Suizhou	Wuhan Huanggang Xianning Huangshi	Jingmen Ezhou Shiyan
2005	Xianning Huangshi Xiaogan Ezhou Wuhan	Jingzhou Yichang Suizhou Xiangyang Huanggang	None	Shiyan Jingmen
2010	Suizhou Jingmen Xiaogan	Yichang Xiangyang Jingzhou	Huanggang Huangshi Wuhan Xianning	Shiyan Suizhou
2015	None	Yichang Xianning Xiangyang Jingzhou Xiaogan Huangshi	Huanggang	Shiyan Jingmen Suizhou Wuhan Ezhou
2019	Huangshi	Xiaogan Huanggang Jingzhou Xianning Xiangyang Yichang	None	Shiyan Jingmen Wuhan Suizhou Ezhou

Based on the gray relational model, this study calculates the driving factors of the coupling coordination degree between UPLSPs and EE in Hubei Province, as shown in Table 10. The relational degree between the coupling coordination degree and the indicators of UPLSPs and EE is basically above 0.5, which indicates the close relationship between them. According to Table 10, the driving factors of coupling coordination in Hubei Province are ranked from strong to weak: total water resources > built-up area > industrial wastewater discharge > number of employees at the end of the period > GDP > industrial SO_2_ emissions > annual electricity consumption > industrial smoke (powder) dust emissions > UPLSPs > total investment in fixed assets in the whole country. This shows that the key factor restricting the coupling coordination of UPLSPs and EE in Hubei Province is the total water resources.

**Table 10:** The correlation between coupling coordination degree and UPLSPs?EE in Hubei Province.

**City**	**X**	**Y_**1**_**	**Y_**2**_**	**Y_**3**_**	**Y_**4**_**	**Y_**5**_**	**Y_**6**_**	**Y_**7**_**	**Y_**8**_**	**Y_**9**_**
Wuhan	0.5999	0.6324	0.8873	0.6792	0.7109	0.8652	0.6760	0.6769	0.6180	0.5947
Huangshi	0.6341	0.6762	0.8266	0.8940	0.8031	0.7435	0.7903	0.8794	0.8043	0.7915
Shiyan	0.7317	0.5213	0.6918	0.7578	0.7091	0.9175	0.6705	0.7217	0.7016	0.6244
Yichang	0.7502	0.4969	0.6328	0.6590	0.5401	0.8136	0.6123	0.6690	0.5743	0.6240
Xiangyang	0.8334	0.5212	0.6437	0.7263	0.6362	0.7775	0.6737	0.7143	0.6255	0.6882
Ezhou	0.6592	0.5252	0.9425	0.8030	0.7136	0.4945	0.6709	0.6953	0.5750	0.6372
Jingmen	0.5922	0.5103	0.6637	0.8649	0.6280	0.8601	0.6555	0.7335	0.7974	0.7323
Xiaogan	0.4328	0.6584	0.7522	0.7892	0.7418	0.8844	0.7593	0.8590	0.8123	0.8975
Jingzhou	0.6129	0.5299	0.7270	0.8723	0.6713	0.9035	0.6864	0.7889	0.6633	0.6810
Huanggang	0.6399	0.5148	0.6011	0.7399	0.5920	0.9322	0.6764	0.7642	0.6518	0.7472
Xianning	0.8470	0.5182	0.6817	0.7598	0.6430	0.8767	0.6713	0.8148	0.6602	0.7900
Suizhou	0.5074	0.6840	0.9294	0.8475	0.7942	0.9067	0.8157	0.8338	0.7409	0.7294
Hubei Province	0.6534	0.5657	0.7483	0.7827	0.6819	0.8313	0.6965	0.7626	0.6854	0.7115

*X is the UPLSPs*.

At the city level, Wuhan, Ezhou, and Suizhou have the highest correlations between the coupling coordination degree and the number of employees at the end of the period, which are 0.8873, 0.9425, and 0.9294, respectively. Huangshi and Jingmen have the highest correlations between the coupling coordination degree with the built-up area: 0.8940 and 0.8649, respectively. Shiyan, Yichang, Jingzhou, Huanggang, and Xianning have the highest correlations between the coupling coordination degree with the total water resource: 0.9175, 0.8136, 0.9035, 0.9035, and 0.8767, respectively. Xiangyang has the highest correlation between the coupling coordination degree and the UPLSPs, equal to 0.8334. Finally, Xiaogan has the highest correlation between the coupling coordination degree and industrial smoke (powder) dust emissions, which is 0.8975.

In view of the factors restricting the coordinated development of UPLSPs and EE in Hubei Province, Wuhan, Ezhou, and Suizhou should promote the orderly flow of labor elements, deepen the reform of the household registration system, smooth the social flow channels of labor and talents, strengthen the introduction of employees, and improve the level of labor market allocation. Huangshi and Jingmen need to pay attention to make full use of the existing land resources, optimize the structure of urban land use and improve the level of the intensive use of land resources. Shiyan, Yichang, Jingzhou, Huanggang, and Xianning ought to highly value regional water resources protection, enhance residents' awareness of environmental protection, prevent water pollution, and promote the rational allocation of water resources, conservation, and management protection. Xiangyang requires leaders to focus on the spatial distribution of the urban population and land. The city is classified as urban population–land relative matching, so a mechanism should be established to assess the spatial pattern of the urban population and land. Xiaogan demands that we strengthen the protection of the ecological environment. Its EE is low, so it should optimize upgrading the industrial structure and take a green and low-carbon development path.

## Discussion

The results of this study showed that from 2000 to 2019 in Hubei Province, UPLSPs exhibited a medium-low mismatch and urban population concentration, and EE was at a medium-low level. The coupling coordination degree between the two was slowly increasing, but it was still low, with strong heterogeneity across regions. The total water resources were the most important factor affecting the coupling coordination between the two. In addition, UPLSPs and EE in Hubei Province had a conflictual relationship, and UPLSPs restricted the development of EE.

First, the UPLSPs of Hubei Province from 2000 to 2019 is classified as urban population concentration, but most cities are considered urban land concentration. There is a phenomenon of urban land concentration transforming to urban population concentration, which is consistent with some previous research (74). This may be because, in the process of urbanization, most local governments have equated urbanization with urban construction, overemphasized the expansion of the urban built-up area, and ignored the implementation of urban population agglomerations and social security after agglomeration, resulting in problems such as the land urbanizing faster than the population. However, to promote the development of new urbanization is essentially to shift from the “land urbanization” mode, which relies on land finance, construction of industrial development zones, and industrial and real estate development in new urban areas, to “population urbanization,” which is oriented to the settlement of the migrating population. Therefore, the evaluation mechanism of urban population–land spatial matching should be established to optimize the flow of elements between the two systems (7, 73). In addition, different measures can be used for different types of cities. For cities that feature an urban land concentration, such as Shiyan, Yichang, Ezhou, Huanggang, Xianning, and Suizhou, we should revitalize the stock of construction land and strictly control its incremental expansion to meet the land demands of urban development and to curb the horizontal expansion of the urban scale. For cities with an urban population concentration, such as Wuhan, Huangshi, Xiaogan, and Jingzhou, we should optimize the upgrading of industrial structure and promote the equalization of public services to meet the land demands of urban population growth and avoid the deterioration of the urban environment through growing traffic congestion caused by increasing limitations on space.

Second, the overall level of EE in Hubei Province is low but with little variation, and there are obvious regional differences, which is consistent with previous research (48). This may be because, with the continuous expansion of the economy and the continuous increase of the population in Hubei Province, the consumption demand for energy resources in the region is increasing, and the production of various pollutants is also increasing, resulting in the increase of ecological pressure and prominent environmental problems; therefore, the level of EE is low. Upgrading the industrial structure should be optimized, and green and low-carbon development needs to be pursued (26). As for cities with high EE, such as Wuhan, Jingmen, and Suizhou, we should actively play the leading role and promote the improvement of EE in surrounding cities by sharing the experiences of ecological environment governance.

Finally, UPLSPs and EE are in conflict, and the UPLSPs of urban land concentration constrains the development of EE, which is consistent with some previous findings (72). This is likely because, with the rapid urbanization of Hubei Province, many people are gathering in cities, urban population density is increasing, and the demand for resources by urban residents have damaged the environment, thus limiting the level of EE. Therefore, it is necessary to strengthen the regional coordination mechanism, clarify the resource endowment advantages and main functions of each city, and vigorously promote the free flow and optimal allocation of production factors within the region (75). Cities with high coupling coordination, such as Suizhou and Wuhan, should give play to the spatial spillover effect, and promote the synergistic development of surrounding cities; cities with low coupling coordination, such as Xiaogan and Huanggang, should transform their development models and take the needs of economic development and the ecological environment into consideration.

The main contributions of this study are several. First, it discusses the dynamic relationship between UPLSPs and EE. Second, the urban population–land spatial matching evaluation model and super-efficient SBM model are constructed to measure UPLSPs and EE, respectively. Finally, the gray relational analysis method is used to analyze the driving factors affecting the coupling coordination degree of UPLSPs and EE in Hubei Province to provide a theoretical reference for regional governments to implement differentiation strategies. However, there are some research limitations. (1) This study constructs the index system of EE based on “input + desired output + undesired output,” but the index selection of the EE input–output system may also contain some other indicators that need to be further explored. This is an aspect that needs detailed research in the future. (2) This study examines the coupling coordination degree between UPLSPs and EE at the city level, and does not investigate the urban agglomeration or county levels. In future research, more in-depth studies need to be carried out along multiple scales and dimensions.

## Conclusion

This study takes Hubei Province as the research area to explore UPLSPs, EE and their degree of coupling coordination in 12 cities from 2000 to 2019. These measures are calculated by constructing a spatial matching evaluation model, super-efficiency SBM model, and coupling coordination degree model. Then, spatial autocorrelation analysis and gray relational analysis methods are used to explore the spatiotemporal evolution characteristics and driving factors of the coupling coordination between UPLSPs and EE. The purpose of this is to guide the rational allocation of urban populations and land and the construction of ecological civilizations in Hubei Province. The results include a few important points. First, the urban population–land spatial matching degree shows a gradual upward trend from 2000 to 2019, but the overall matching level is not high; the average value of EE takes an “N”-shaped trajectory, and its overall level is low, with obvious regional differences. Second, the average value of the coupling coordination degree between UPLSPs and EE exhibits a slow upward trend, with a radial distribution such that levels are high in the middle and low along the periphery. There is a conflictual relationship between UPLSPs and EE, and the former restricts the development of the latter. Finally, there is a strong correlation between the degree of coupling coordination and various indicators of UPLSPs and EE.

## Data Availability Statement

The raw data supporting the conclusions of this article will be made available by the authors, without undue reservation.

## Author Contributions

XC designed the research framework. GZ revised the whole paper. LS analyzed the data and written the main sections. LS, YJ, CL, and JZ collected the data and discussed the results. All authors read and approved the final manuscript.

## Funding

The Fundamental Research Funds for the Central Universities, Zhongnan University of Economics and Law (CN) (NO. 2722022BY014); the Hubei Provincial Department of Education Project of Philosophy and Social Sciences (CN) (No. 21G015).

## Conflict of Interest

The authors declare that the research was conducted in the absence of any commercial or financial relationships that could be construed as a potential conflict of interest.

## Publisher's Note

All claims expressed in this article are solely those of the authors and do not necessarily represent those of their affiliated organizations, or those of the publisher, the editors and the reviewers. Any product that may be evaluated in this article, or claim that may be made by its manufacturer, is not guaranteed or endorsed by the publisher.
